# Nephrology providers’ perspective and use of mortality prognostic tools in dialysis patients

**DOI:** 10.1186/s12882-024-03861-y

**Published:** 2024-11-26

**Authors:** Jennifer Bergeron, Christina Marchese, Colton Jensen, Sean Meagher, Amanda G. Kennedy, Bradley Tompkins, Katharine L. Cheung

**Affiliations:** 1 Division of Nephrology, Department of Medicine, West Virginia University School of Medicine, 1 Medical Center Drive, PO Box 9165, Morgantown, WV 26506 USA; 2 Division of Nephrology, Department of Medicine, The University of Vermont Medical Center, Burlington, VT USA; 3 Tufts University School of Medicine, Boston, MA USA; 4 Department of Medicine, Beth Israel Deaconess Medical Center, Boston, MA USA; 5 The Robert Larner, MD College of Medicine at The University of Vermont, Burlington, VT USA; 6 Department of Medicine Quality Program, The Robert Larner, MD College of Medicine at The University of Vermont, Burlington, VT USA; 7 The Center On Aging, Larner College of Medicine at The University of Vermont, Burlington, VT USA

**Keywords:** Mortality, Tools, Prognostication, Dialysis, Kidney failure, Qualitative, Perspectives

## Abstract

**Background:**

Mortality prognostic tools exist to aid in shared decision making with kidney failure patients but are underutilized. This study aimed to elucidate nephrology providers’ practice patterns and understand barriers to prognostic tool use.

**Methods:**

Nephrology providers (8 physicians and 2 nurse practitioners) at an academic medical center underwent semi-structured interviews regarding their experience and perspective on the utility of mortality prognostic tools. Common themes were identified independently by 2 reviewers using grounded theory. Three six-month mortality prognostic tools were applied to the 279 prevalent dialysis patients that the interviewed providers care for. The C statistic was calculated for each tool via logistic regression and subsequent ROC analysis. Nephrology providers reviewed the performance of the prognostication tools in their own patient population. A post interview reassessed perspectives and any change in attitudes regarding the tools.

**Results:**

Nephrology providers did not use these mortality prognostic tools in their practice. Key barriers identified were provider concern that the tools were not generalizable to their patients, providers’ trust in their own clinical judgement over that of a prognostic tool, time constraints, and lack of knowledge about the data behind these tools. When re-interviewed with the results of the three prognostic tools in their patients, providers thought the tools performed as expected, but still did not intend to use the tools in their practice. They reported that these tools are good for populations, but not individual patients. The providers preferred to use clinical gestalt for prognostication.

**Conclusion:**

Although several well validated prognostic tools are available for predicting mortality, the nephrology providers studied do not use them in routine practice, even after an educational intervention. Other approaches should be explored to help incorporate prognostication in shared-decision-making for patients receiving dialysis.

**Supplementary Information:**

The online version contains supplementary material available at 10.1186/s12882-024-03861-y.

## Introduction

Kidney disease is common and highly morbid, with over 3 million people worldwide receiving dialysis. The mortality rate among patients receiving maintenance dialysis is a staggering 60% at 5 years [1]. However, much heterogeneity exists [1], making it difficult to predict patients’ outcomes, particularly in older adults [2]. Accurately predicting mortality is essential for prognostication and honest conversations may enhance advance care planning. In fact, studies have shown that patients with chronic and end stage kidney disease desire this prognostic information in shared decision making (SDM) [3–7]. In addition, the ASN Choosing Wisely Campaign [8], the RPA Clinical Practice Guidelines [9], and the KDIGO 2012 CKD guidelines [10] support that individualized prognostic information should be included in the decision to initiate dialysis.

Because prognostication is challenging, several prognostic tools have been developed to help make an accurate prognosis that can be used in these conversations. However, a recent study of Canadian nephrology providers found that > 80% of providers use clinical gestalt to prognosticate and 70% never or rarely use clinical prediction tools [11]. To our knowledge, there is little research focusing on Nephrology providers’ perspectives about and method of use of these tools in the real world.

This study aimed to elucidate Nephrology providers’ attitudes about and practice patterns of mortality prognostic tools in their care of patients on dialysis. This study also aimed to discover whether their perspectives and use of these tools changed after they were presented with data on how these tools performed in their own patients and the patients in their state.

## Methods

### Study setting and participants

This study was conducted at the University of Vermont Medical Center (UVMMC), located in Burlington, Vermont. UVMMC is Vermont’s only academic medical center and serves over 1 million patients in Vermont and northern New York. There are six UVMMC affiliated, non-profit dialysis units. All Nephrology providers (8 physicians and 2 nurse practitioners) caring for patients receiving maintenance dialysis were eligible to participate.

### Qualitative study methods

Semi-structured interviews were conducted via Zoom (Zoom Video Communications, Inc., San Jose, CA) by the first author (JB), in May 2020. Two of the Nephrology providers had worked with JB (medicine resident) before as the attending on Nephrology consults. All the providers knew JB and knew that she was doing this project to support her application to nephrology fellowship. Providers were asked about their knowledge of and experience with mortality prognostic tools for patients receiving dialysis (see interview guide—Supplement 1). The interviews were 20 min ± 10 min. No field notes were made. The interviews were recorded and transcribed verbatim by CM and JB. The transcripts were not returned to the participants for comment or correction.

### Qualitative study analysis

Two members of the study team, the principal investigator (JB) and a medical student who did not know any of the providers (CM) performed a thematic analysis for content using the transcripts. The backbone of the code tree was created using the questions from the semi-structed interview guide, but the data for each question was analyzed using grounded theory. The initial codes were generated independently and then they were reviewed together for each interview and themes were identified. Disagreements about themes, the coding tree, and final coding were resolved by discussion.

### Mortality prognostic tool selection

Three mortality prognostic tools commonly reported in the literature and available without cost were selected (See Table 1). Cohen et. al’s 2010 model was derived from 514 prevalent hemodialysis patients in New England using age, albumin, dementia, peripheral vascular disease and the surprise question: “Would I be surprised if this patient died in the next six months?” [12]. Charlson et al.’s “Charlson Comorbidity Index” (CCI) was derived from 559 medical patients in the US using age and 16 comorbidites [13]. Couchoud et. al’s algorithm in 2015 was derived from 24,348 incident elderly ESKD patients over 75 years old in France using age, gender, albumin, five comorbidities, and mobility [14]. The three prognostic tools were chosen because they focus on different aspects of prognostication: Cohen’s tool includes provider gestalt with use of the surprise question, Charlson is heavily weighted by comorbidities and is the most commonly used prognostic tool [15], and Couchoud tool includes mobility and was designed to be used in older adults.

**Table 1:** Mortality prognostic tools, and their required data elements, selected for this study

	Cohen	Charlson	Couchoud
Age	X	X	X
Gender			X
Albumin	X		X
Comorbidities	Dementia, peripheral vascular disease	Myocardial infarction, congestive heart failure, peripheral vascular disease, dementia, COPD, connective tissue disease, diabetes, hemiplegia, chronic kidney disease, solid tumor, lymphoma, leukemia, AIDS	Congestive heart failure, peripheral vascular disease, dysrhythmia, active cancer, severe behavioral disorder
Mobility			X
Surprise Question (Would I be surprised if this patient dies in the next six months)	X		

### Mortality prognostication and measurement

In April 2020, 279 prevalent dialysis patients cared for by these Nephrology providers were identified and prospectively followed for six months. All patients receiving maintenance dialysis were included. Data were extracted through chart review of the dialysis electronic medical record (CyberRen) and UVMMC’s EMR (EPIC) in April 2020. Most patients had data in both EMRs. A standardized approach to identify comorbid conditions from the EMRs was used. To capture the most complete assessment of burden of comorbid conditions, a patient was considered to have a comorbidity if it was listed in at least one of the EMRs (as problem list completeness in EMRs varies anywhere from 60–99% [16]). A patient was considered to have the more severe disease stage if the stages differed in the two EMRs. The most recent serum albumin resulted before May 1st, 2020 was chosen.

The providers were given a list of their patients and asked to answer the surprise question [12] for each. The responses and patient characteristics were used in the corresponding online calculators for the prognostic tools [17–19]. Each patient had a score calculated for each of the three tools (Cohen’s result was a percentage from 0 to 100, Charlson’s was a score from 0 to 37, and Couchoud’s was a score from 0 to 28).

At six months follow up, EMR review was used to identify patients who had died. The C statistic, or discrimination, for each tool was calculated via logistic regression and subsequent receiver operating characteristic (ROC) analysis using Stata (Stata 16.1, Stata Corp, LLC. College Station, TX). A C statistic of 0.5 is no better than flipping a coin, 0.7 is considered a good model and a C statistic of 0.8 is considered a “strong” or “excellent” model.

### Brief intervention and follow up interviews

A similar process of email invitation, semi-structured interview (Supplement 2), transcription, and coding was used for the follow up interviews. Providers received the results of the prognostic tools, patients’ outcome at the time of the email invitation, and the percentage of their own accuracy with the surprise question (Supplement 3). Results were also reviewed with the providers at the beginning of the interview before the follow-up questions were asked. The follow up interviews were shorter, on average 10 min ± 5 min.

## Results

The providers (8 MDs and 2 NPs) who participated in the study were 50% female, 60% Caucasian, 30% Asian, and 10% Black and had a mean age of 54 (range 36–73). They had an average of 17 years of practice (range 2 years to 43) and had been trained in a wide variety of locations. They each cared for an average of 34 patients (range 6–55).

### Providers’ views on mortality prognostic tools

Providers were only aware of 2 tools to predict mortality in dialysis patients. 80% of the providers had heard of Cohen’s mortality prognostic tool, especially regarding the surprise question. 10% of the nephrology providers had heard of Charlson comorbidity index. None of the nephrologists used these tools in their current practice.

Representative quotes of providers’ views on mortality prognostic tools can be seen in Table 2. The main barrier identified to use was provider concern that the tool was not applicable or accurate in their specific patients. Most providers also noted that the disease course itself is unpredictable. Time restraints and the addition of more “work” was a barrier identified by all the providers. Lack of knowledge of the tools and the data behind them were also acknowledged by 6 of the 10 providers. All the providers identified clinical experience as their main source of prognostication.

**Table 2:** Nephrology providers’ perspective on the barriers to use of mortality prediction tools

Tools are not applicable or accurate in their patients	*“Of course, with any calculator, there’s going to be variability. Some people might live longer than what the calculator predicted, some less.”* *“I don’t think I’ll live long enough to see a predictive system that I will really believe is that accurate in terms of predicting how people will do.”* *“I see a big problem with all risk assessment tools. Not that the variables themselves are not valid, but how are they weighted in terms of driving the end number.”* *“What are the barriers to using the tool? The first one is believing it.”* *“I’ve never used it again for predicting for particular person because I don’t think one can be that certain.”*
Disease course is unpredictable	*“Sometimes I’m surprised that there are people who don’t show up for dialysis for weeks, are non-compliant with medications, and they do fine. While some people who follow all the rules suddenly die. It feels unpredictable.”* *“I worry more that people want to try to develop tools to give certainty, when I know there really isn’t any.”*
Time restraints and the addition of more “work”	*“It takes time.”* *“Is it just adding more burden, adding more noise without telling you much.”* *“I’m sitting with a patient and, oh yeah, I’ve got this tool and oh my goodness, I can’t remember where I where I’ve hidden it and it’s somewhere in the computer, but I don’t know which one it’s in and isn’t in. And has it been calculated and what numbers do I have to put into it. Where do I find those numbers. And then I have to do all that. And for somebody who’s technologically challenged like me that gets put to the side really quickly.”* *“Actually stopping to take the time to import it into a tool doesn’t seem very helpful for me, especially since I know these patients well.”*
Lack of knowledge	*“Well, number one, not knowing about it.”* *“I don’t know how hard it is [to use] honestly.”*
Clinical experience as the main source of prognostication	*“I don’t want to quantitate those sorts of things because I just think about it.”* *“When you’ve done this for a number of years, your clinical experience helps you out here.”* *“I guess I’m looking at the same points, but not putting it in the calculator.”* *“So I’m able to make that judgment without resorting to the tool.”*
Uncertainty if mortality prognostic information would change patients’ decisions	*“It would give us an idea of who would do badly or not, but I know that doesn’t change the fact that if patients want to continue, they will continue.”* *“But in the end, it still depends on the patient’s wishes, whether they want to try something.”* *“I’m not going to say you can’t do it just because you would do poorly, so it gives us an idea of you know how to talk to them and which way we should probably lean towards but it’s still you know their choice.”* *“People don’t make decisions based on that information very often. They make decisions based on whether they are risk averse or risk tolerant.”* *“It’s mostly what do people want, what do the people around them want?”*

The providers identified a few advantages to using mortality prognostic tools. They noted that some patients are number-oriented and being able to provide that information may help those patients in decision making. Providers also noted that these prognostic tools, if predicting a poor prognosis, would be a reminder to have a goals of care conversation and make providers more likely to encourage supportive care over dialysis.

The majority of the providers reported they were open to the idea of using these tools in their prognostication if further evidence for the validity and education about the use of these tools was provided. Providers noted that if a tool was shown to have strong discrimination and predicted a high mortality, it would change how they discuss management options with the patient- i.e. make them more likely to encourage supportive care over dialysis. At the same time though, providers expressed that mortality was not the key factor on which to prognosticate and that patients will make decisions based on a variety of quality of life measures.

### Validation of mortality prognostic tools

**Fig. 1:**
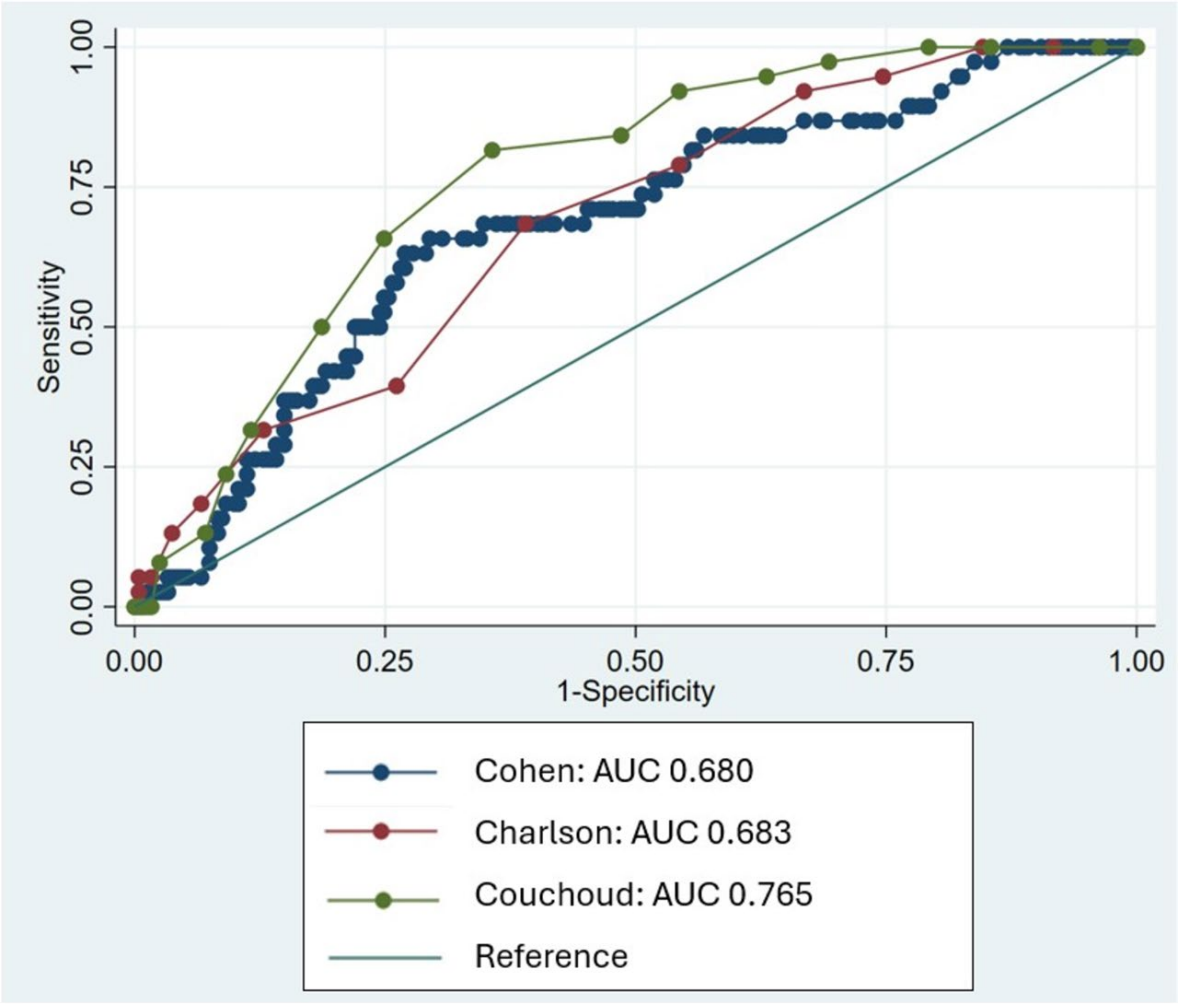
Comparison of receiver operating characteristics curves for predicting 6-month mortality for UVMMC patients on dialysis among the Cohen, Charlson, and Couchoud tools

The overall 6-month mortality in Vermont’s prevalent dialysis population was 14%. Couchoud had the best discrimination of 6-month mortality in Vermont’s dialysis patients with a C statistic of 0.77 compared to Cohen and Charlson at 0.68 (Fig. 1).

### Prognostication accuracy of providers

The accuracy of each provider’s response to the surprise question varied across provider. The average accuracy of providers was 68%, range 47–91%.

### Post intervention interview

Five physicians and two nurse practitioners participated in the follow up interviews as of March 2023. Of the remaining three physicians, one no longer worked at the study site, one was on maternity leave, and one did not respond to emails to arrange a second interview.

The providers overall thought the tools performed about “as well as expected.” *“There were no surprises.” “I think they’re about what you would expect because I’ll never be that excited about predictive tools.”* This perception did not vary between clinicians whose answers to the surprise question were more or less accurate.

Providers speculated that the Couchoud model performed the best because of its inclusion patient mobility and tied that in with frailty as a risk factor for not doing well on dialysis.

“I do think that mobility is a major factor for a lot of patients, so I do think that it was a good idea for the Couchoud model to include that.”

“I like these, these factors in this tool, with the albumin-nutrition and the frailty, because I know those are independent predictors of those not doing well on dialysis.”

“It actually makes me think more of mobility as an important index of patient wellness.”

Though some acknowledged that it may be because the original Couchoud cohort had the largest study population.

Still, while most providers endorsed a “role” for using risk assessment tools, none of the providers routinely used the tools or had plans to implement it into their practice.

Providers again voiced concern that although tools are good for populations, and even their specific population, but that they were not accurate for any one specific patient.

“I think the tools are reasonably good at predicting what will happen in the population, not particularly for what will happen in an individual. So obviously that makes the utility of that somewhat questionable when you’re dealing with the individual rather than planning for the population.”

“I mean they’re nice for studies if you are trying to look at large populations or and you have to have a particular reason for wanting to understand that particular prediction. But for individuals they’re never terribly good so I’m not totally surprised.”

Providers still identified clinical experience and gestalt as their main determinants of prognostication. This was true for all providers, regardless of their own accuracy with answering the surprise question. A few providers noted that after seeing this data, they might try to incorporate some of the individual risk factors from the tools into their clinical assessment. “*I would place it in my ‘subjective-ometer’ when I’m thinking about these things with the patient.”*

## Discussion

This study assessed nephrology providers’ perspectives and use of mortality prognostic tools in dialysis patients. We found that nephrology providers had some knowledge of prognostic tools but did not routinely use them in practice. The main barrier identified to using prognostication tools was the perspective that they are not generalizable nor specific enough for a given patient. After external validation of three routinely available prognostic tools in these providers’ practice, and demonstration that these tools performed better than clinician’s gestalt, perspectives were seemingly unchanged reflecting a lack of trust in using mortality prognostic tools. Time constraints were also a theme throughout this study, as there was also interest in the tools automatically generating prognostic information in the EMR and finding that Cohen’s tool, the tool with the least number of input variables, was the tool the providers had heard of or used the most. Provider interviews suggested a need for better training on how to incorporate prognostic information into serious illness conversations with patients.

Several prognostic tools for mortality on dialysis exist, but few have been externally validated [20]. This study revealed that generalizability was a major concern for nephrology providers. The Vermont population is predominantly rural, white, older, and of a lower socioeconomic status than the derivation cohorts and other studies have shown that these tools don’t perform well in older populations [2]. To respond to this concern, three prognostic tools were validated in providers’ dialysis patient population. In this study, Couchoud’s tool had the highest discrimination for six month mortality with a C statistic of 0.765, which is comparable to the highest C statistic found in the 2019 meta-analysis of 32 indices to predict mortality in incident dialysis patients (C statistic 0.74) [15]. There, the overall C statistic was 0.71 for any prediction length for mortality and had high heterogeneity, with the sub group analysis for models predicting six month mortality range having a C statistic of 0.540–0.896. It is worth noting that the metanalysis was in incident patients rather than the prevalent population in our study. The current study showed that Couchoud’s tool had strong discrimination for six mortality and should have assuaged providers’ concerns about external validation, allowing other barriers to be identified in the follow up interviews.

Our study confirmed findings by Forzley et al [11] that nephrology providers do not use prognostic tools to provide prognostic information, preferring clinical gestalt. In addition, this study demonstrated that provider preference did not change even after validation of the tools in their patients or observing that their clinical gestalt had slightly lower accuracy than that of the prognostic tools. Therefore, creating more accurate prognostic tools or making them easier to implement may *not* increase providers’ use. Provider perspectives suggest a disconnect in patient-physician communication around prognosis, as providers report they are comfortable using gestalt to prognosticate, but other studies show patients aren’t receiving the prognostic information they desire [3, 5, 6]. This suggests that helping providers to refine the accuracy of their clinical gestalt and convey it more effectively to patients may be of higher utility to improve prognostic communication. One such example is a recent pilot study that found training nephrologists to use best case/worst case communication improved SDM about dialysis and may increase access to palliative care [21]. Even as far back as 2016, Couchoud et al. called for other prognostic markers [22]. Interestingly, providers in our study self-identified that Couchoud’s tool may have been the most accurate due to the mobility factor and that they would like to use that factor to refine their prognostication. As more evidence mounts that dialysis does not confer morbidity or mortality benefits for all patients with kidney failure [23, 24], future studies are needed to help bridge this prognostication gap.

This study adds to the growing identification of systems issues preventing optimal advance care planning [25, 26]. We describe that although providers felt comfortable with these conversations, they also reported a clear inability to embrace principles of SDM as they felt that the final decision always rests with the patients (Table 2). SDM is key in these situations and is recommended in our clinical practice guidelines [10], but providers receive little training for SDM in fellowship or practice [27, 28]. It is possible that providers feel ill-equipped or overwhelmed in these patient focused conversations, as kidney disease care, especially dialysis, is inherently “disease oriented” [29]. Studies are beginning to look towards leveraging all members of the interdisciplinary dialysis team to promote advance care planning in patients on dialysis [30, 31]. Systems based approaches are needed to facilitate learning and skill building to create individualized care plans with patients and their families living with kidney failure.

This study, as the first to evaluate Nephrology providers’ perceptions and barriers to use of mortality prognostic tools, had several strengths. Foremost was this study’s use of mixed methods and a brief intervention. Externally validating the tools addressed a major concern that the providers identified in the first interview and allowed the subsequent interviews to capture other unresolved barriers. Furthermore, giving the providers the results of their patients’ 6-month mortality next to their predictions and the predictions from the tools (Supplement 3) yielded more grounded and real-world discussion of their perceptions. Performing the second interviews allowed for analysis of any dynamic perceptions and verified previous themes, which is often not done in qualitative studies. Lastly, the choice of prognostic tools with different aspects of prognostication allowed the interviews to capture provider perspectives on which parts of prognostication are highest yield.

There were limitations to this study. First, it is a small sample size of both providers and patients from one state, and not all providers were available for the second interview. Therefore, the interviewed providers’ responses may not be generalizable. However, the providers do have a wide variety of training backgrounds, employment history, and practice length. Second, social acceptability bias may have been at play as the interviews were not blinded and the primary author conducting the interviews was a resident interested in Nephrology at their institution. Third, the interviews were semi-structured which gave the opportunity for more in-depth conversation, but may have introduced interviewer bias with leading questions, wording bias, or confirmation bias. Lastly, the study was conducted during the COVID-19 pandemic which could have contributed to the observed mortality, but Vermont had < 15 COVID-19 related deaths during our study period [32, 33], and only 2% of covid-cases at that time were reported to have “chronic kidney disease” [34] making it unlikely that this skewed the results of our data.

In conclusion, several well validated prognostic tools are available for predicting mortality in dialysis patients, but nephrology providers do not use them in routine practice due to concerns about their applicability in their patients. Addressing the barriers of external validity and lack of knowledge of the tools did not change the nephrology providers’ use or attitude towards the tools. Implementation research is needed to help providers share prognosis and enhance shared-decision-making surrounding dialysis.

## Supplementary Information

Supplementary Material 1.

## Data Availability

The dataset generated and/or analyzed during the current study are not publicly available due to this research being considered exempt and we do not have consent from participants to share raw data. Deidentified data may be available from the corresponding author on reasonable request.
