# Supplementary material for: Nephrology providers’ perspective and use of mortality prognostic tools in dialysis patients
Source: BMC Nephrol. 2024 Nov 26;25:425. doi: 10.1186/s12882-024-03861-y (PMC11590527; doi:10.1186/s12882-024-03861-y)
Supplement: Supplementary file 1 — Supplementary Material 1. [file 12882_2024_3861_MOESM1_ESM.docx]

**Supplement 1.** Interview guide for initial interview.

1. Tell me about your use of risk assessment tools in prognostication.
   1. Which tools do you use?
   2. How do you use the tool? (Do you show the patient the number, use it in shared decision making, or use it for your own assessment?)
   3. When do you use these tools? Initiating dialysis, coming off dialysis, etc.
   4. What tools do you use / are you aware of any tools for other outcomes (e.g. function, symptoms)
2. What are the advantages to using a mortality risk assessment tool?
3. What are the barriers to using a mortality risk assessment tool?
4. How does using a mortality risk assessment tool affect your management?
   1. Should a mortality risk assessment tool affect your management?

**Supplement 2.** Interview guide for follow up interview.

1. What do you think of the performance of these tools?
2. Did the tools perform as expected? If not, what do you think were the contributing factors?
3. Do you see a role in using a risk assessment tool in prognostication?
4. Is this data going to change how you use or don’t use risk assessment tools?
5. After reviewing this data, would you be more likely to offer supportive/conservative management?

**Supplement 3.** Summary data presented to nephrology providers as brief intervention prior to follow up interview.

**
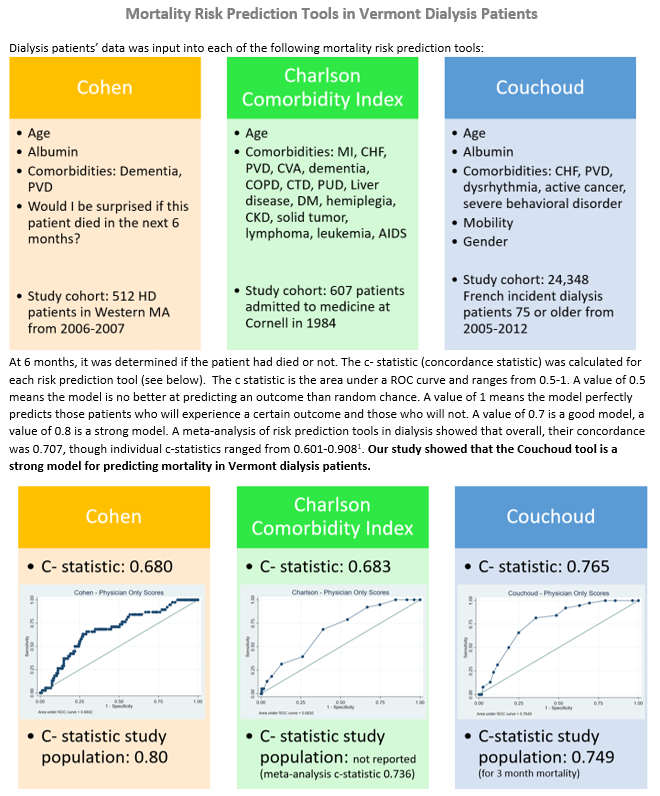
**

**
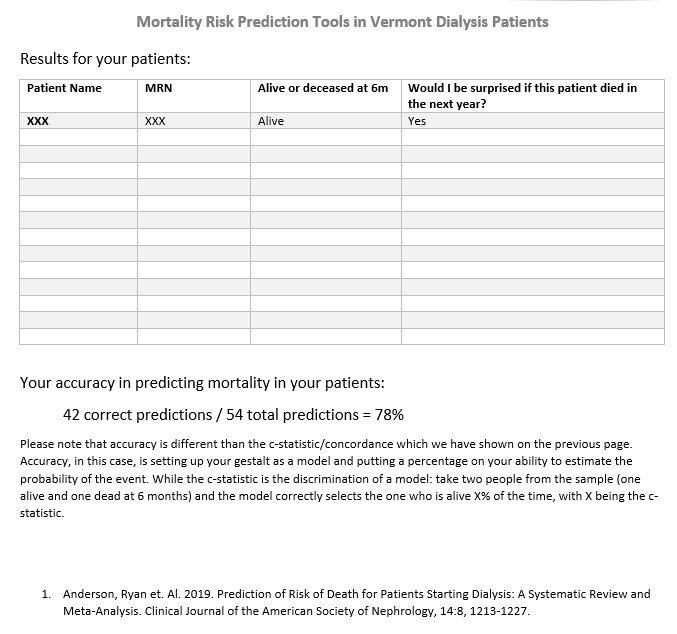
**
